# Supplementary material for: Content-rich biological network constructed by mining PubMed abstracts
Source: BMC Bioinformatics. 2004 Oct 8;5:147. doi: 10.1186/1471-2105-5-147 (PMC528731; doi:10.1186/1471-2105-5-147)
Supplement: Additional File 2 — The original results of the above study (non-essential files are deleted to keep the file size under the limit set by BMC bioinformatics). [file 1471-2105-5-147-S2.bz2 › chilibotAdditionalFile2/dip20/399ID9662041E9953/html/EGFR_BTC.html]

 


 **EGFR** and **BTC** 
  
Found 65 abstracts in PubMed, retrieved 20.  
 

 What does Google say? 
 PDF only 
| .edu only 

---

**Interactive relationship** (e.g. stimulation, inhibition, etc)

**Stimulatory relationship**- The results are consistent with a model in which GLP 1 increases PI 3 kinase activity and enhances beta cell proliferation via transactivation of the  **EGFR**  that would require the proteolytic processing of membrane anchored  **BTC**  or other EGF like ligands.  Ref: 12502502 Diabetes, 2003
- Recombinant rat  **BTC**  stimulated the proliferation of mouse Balb c 3T3 fibroblasts and competed for binding to the ErbB1  [ **EGFR** ]  receptor in a dose dependent manner analogous to that of  **BTC**  purified from natural sources.  Ref: 11564606 J Mol Endocrinol, 2001
**Neutral relationship**- Heparin binding epidermal growth factor HB EGF and betacellulin  [ **BTC** ]   **BTC**  are activating ligands for EGF receptor  **EGFR**  ErbB1  [ **EGFR** ]  and ErbB4.  Ref: 12773386 EMBO J< MedlineTA>EMBO J, 2001
- **BTC** , acting through EGF R erbB 1  [ **EGFR** ] , is important for the differentiation of beta cells.  Ref: 12399441 Endocrinology, 2002
- Betacellulin  [ **BTC** ]  and heregulin NDF alpha strikingly colocalized with  **EGFr**  and erbB3 receptors in the granular layer and in a declining gradient from the granular zone to the basal layer, respectively.  Ref: 12768307 Arch Dermatol Res< MedlineTA>Arch Dermatol Res, 2002
- Betacellulin  [ **BTC** ]   **BTC**  belongs to the epidermal growth factor EGF family of peptide ligands that are characterized by a six cysteine consensus motif EGF motif that forms three intra molecular disulfide bonds, crucial for binding the ErbB  [ **EGFR** ]  receptor family.  Ref: 12031500 Biochim Biophys Acta, 2002

**Non-interactive relationship** (e.g. studied together, co-existance, homology, etc.)

- Little is known about the regulative role of the four other widely expressed epidermal growth factor receptor  [ **EGFR** ]  ligands, heparin binding EGF, amphiregulin, betacellulin  [ **BTC** ]  and cripto in the gastric mucosa.  Ref: 11218236 Scand J Gastroenterol, 2001
- This hypothesis was supported by the finding that the growth inhibitory effect of marimastat was directly related to its ability to prevent the release of major c erbB  [ **EGFR** ]  ligands including transforming growth factor alpha, betacellulin  [ **BTC** ]  and heregulin beta1 from HNSCC.  Ref: 12124801 Int J Cancer, 2002
- Moreover, immunohistochemical staining revealed that that  **BTC** , ErbB1  [ **EGFR** ] , and ErbB4 are upregulated in the plaques of human atherosclerotic coronary arteries.  Ref: 12869389 Circ ResCirc Res, 2002
- **BTC**  and EP also present the distinctive feature that they activate all possible heterodimeric ErbB  [ **EGFR** ]  receptors.  Ref: 12111392 J Mol Model , 2002
- Using semi quantitative reverse transcription PCR, we showed that all three c erbB  [ **EGFR** ]  ligands up regulated VEGF A mRNA all isoforms and VEGF C  **BTC**  max at 1 10 nM.  Ref: 11235991 Clin Exp Metastasis, 2000
- ICR62 also reversed the effects of all three erbB  [ **EGFR** ]  ligands TGF alpha,  **BTC**  and HRG beta1 on VEGF A, VEGF C and VEGF D expression.  Ref: 11235991 Clin Exp Metastasis, 2000
- Genomic structure and promoter characterization of the gene encoding the ErbB  [ **EGFR** ]  ligand betacellulin  [ **BTC** ] .  Ref: 12031500 Biochim Biophys Acta, 2002
- Both the c Src inhibitor PP1 and the  **EGFR**  specific inhibitor AG1478 blocked GLP 1 induced 3 H thymidine incorporation in INS 832 13 cells as well as in isolated rat islets, while only AG1478 inhibited the proliferative action of betacellulin  [ **BTC** ]   **BTC** , an  **EGFR**  agonist.  Ref: 12502502 Diabetes, 2003
- A soluble erbB  [ **EGFR** ]  4 binding fusion protein totally inhibited the effects of NRG 4 but not of  **BTC** .  Ref: 12399441 Endocrinology, 2002
- Pretreatment with effective concentrations of ErbB1  [ **EGFR** ]  inhibitor did not suppress  **BTC**  induced kinase phosphorylation.  Ref: 12475887 FASEB J, 2003
- We then explored the regulatory roles of three major ligands with different selectivity of binding to c erbB  [ **EGFR** ]  receptors namely transforming growth factor alpha TGF alpha, betacellulin  [ **BTC** ]   **BTC**  and heregulin beta1 HRG beta1 on VEGF A, B, C and D expression in selected HNSCC lines.  Ref: 11235991 Clin Exp Metastasis, 2000
- **BTC**  induced phosphorylation of all four EGF receptors present on HTASMCs ErbB1  [ **EGFR** ] , ErbB2, ErbB3, and ErbB4.  Ref: 12869389 Circ ResCirc Res, 2000
- The data herein demonstrate that heregulin or betacellulin  [ **BTC** ] , but not EGF, promotes the rapid translocation of ErbB  [ **EGFR** ]  2, the heterodimerization partner for ErbB  [ **EGFR** ]  4, to the same detergent insoluble fraction in T47 D mammary carcinoma cells.  Ref: 11439355 Oncogene, 2001
- Reverse transcription polymerase chain reaction was performed on each sample with primers for rat epidermal growth factor, epidermal growth factor receptor  [ **EGFR** ]  ErbB  [ **EGFR** ] , heparin binding epidermal like growth factor, hepatocyte growth factor, hepatocyte growth factor receptor, keratinocyte growth factor, betacellulin  [ **BTC** ] , amphiregulin, and neuregulin alpha.  Ref: 12148846 Laryngoscope, 2002
